# Low Vitamin D Status Is Associated with Increased Risk of Mortality in Korean Men and Adults with Hypertension: A Population-Based Cohort Study

**DOI:** 10.3390/nu14091849

**Published:** 2022-04-28

**Authors:** Dahyun Park, Juhee Lee, Clara Yongjoo Park, Min-Jeong Shin

**Affiliations:** 1 Interdisciplinary Program in Precision Public Health, Graduate School, Korea University, Seoul 02841, Korea; ekgus7171@korea.ac.kr (D.P.); jhlee1109@korea.ac.kr (J.L.); 2 Department of Food and Nutrition, Chonnam National University, Gwangju 61186, Korea; 3 School of Biosystems and Biomedical Sciences, College of Health Science, Korea University, Seoul 02841, Korea

**Keywords:** vitamin D, mortality, cancer, cardiovascular disease, hypertension

## Abstract

Background Recent randomized controlled trials (RCTs) have shown no effect of vitamin D supplementation on cardiovascular disease, cancer events and mortality or all-cause mortality in Western populations. However, there has been a lack of research on populations with low vitamin D status, including Asians. In addition, there have been indications that an individual’s sex or hypertension status may affect the relationship between vitamin D status and mortality. In this study, we retrospectively assessed the association between vitamin D status and all-cause, cardiovascular, and cancer mortality in Koreans using a national database, and stratified participants according to sex and hypertension status. Methods Participants in the Korean Health and Nutrition Examination Survey 2008–2014, who consented to their data being synthesized with mortality data (up to December 2019), were included (n = 22,742; mean follow-up: 8.9 years). Participants’ level of serum 25-hydroxyvitamin D (25(OH)D) was measured by radioimmunoassay and categorized as <12, 12–19.9, and ≥20 ng/mL. A Cox proportional hazard model was used to assess the risk of mortality. Results In the total sample, risk of all-cause, cancer, and cardiovascular mortality was greater in adults with a serum 25(OH)D level below 12 and 12–19.9 ng/mL than those with ≥20 ng/mL. Men and adults with hypertension, who had low vitamin D status, had a higher risk of cancer and cardiovascular mortality, but not women or adults without hypertension. Similar results were observed when various cutoffs for 25(OH)D were employed, or extrinsic deaths were excluded. Conclusions Vitamin D status below 20 ng/mL is associated with a higher risk of mortality in Korean adults, especially in men and those with hypertension, on the basis of data from a nationally representative sample. Further RCTs on Asian adults with low vitamin D status are warranted.

## 1. Introduction

Cardiovascular disease (CVD) and cancer are the leading causes of death worldwide, including both in the US and Korea [1,2,3]. Recently, the number of deaths per 100,000 of population due to these diseases has been declining in the US but increasing in Korea [3,4]. On the other hand, the prevalence of vitamin D deficiency has declined in the US but increased in Korea during the past few decades [5,6]. Vitamin D deficiency has been associated with a higher risk of cardiovascular events and cancer in many epidemiologic studies [7,8,9]. Mechanisms by which vitamin D may maintain cardiovascular health and prevent cancer have been proposed [8]. However, the results of randomized controlled trials (RCTs) point to a lack of effect of vitamin D supplementation on cardiovascular events, cancer, and mortality [10,11,12,13,14,15,16,17]. These results may be due to the small supplemented dose received by, and the high baseline vitamin D status of, the participants in these studies (mean level of serum 25-hydroxyvitamin D [25(OH)D] ≥ 25 ng/mL) [10]. Investigation of subjects with lower vitamin D status is required to fully understand if a certain threshold of vitamin D must be attained to prevent CVD, cancer and related mortality. In addition, hypertension and an individual’s sex are risk factors for CVD and possibly for certain cancers [18,19,20], but the interaction of hypertension or an individual’s sex with vitamin D status and mortality has not been investigated, especially in populations with relatively low vitamin D status.

Asians are considered to be vitamin D-deficient despite the lack of evidence of the adequacy or otherwise of vitamin D status in Asians. Asians have lower mean 25(OH)D compared to Caucasians, but whether this results in physiological deficiency has not been studied through RCTs on health outcomes, including mortality. Among the few RCTs conducted in Western countries, Asians comprised only a small percentage of the samples, or none at all [13,14,15], and no RCTs have been performed in Asia. The results of nested-cohort studies on Japanese and Chinese participants on the association between vitamin D status and cancer incidence are mixed, while no relationships have been found for all-cause and cardiovascular mortality in a Mendelian randomization or a prospective follow-up study, both performed with Chinese participants [21,22,23,24,25,26,27]. Recently, cause of death data were correlated with data from the Korea National Health and Nutrition Examination Survey (KNHANES), a national database, enabling us to retrospectively examine the association between vitamin D status and mortality in Koreans. Therefore, we tested the hypothesis that low vitamin D status is associated with higher risk of mortality, including mortality caused by cardiovascular issues and cancer, in Koreans. We also investigated the risk of mortality according to vitamin D status stratified by sex or hypertension status.

## 2. Materials and Methods

### 2.1. Study Population

In this study, data from the Korea National Health and Nutrition Examination Survey and Cause of Death data (v1.2) were synthesized. Data from the years 2008–2014 compiled by KNHANES, a nationally representative annual cross-sectional survey conducted by the Korea Disease Control and Prevention Agency (KDCA) [28], were compared with Cause of Death data, which are available from Statistics Korea. KNHANES participants were selected for this study using a stratified, multistage probability cluster sampling method based on sex, age, and geographic area. With the consent of the chosen participants, the KNHANES data were compared with mortality records up to 31 December 2019. This survey was approved by the Ethics Committee of the KDCA (2007-02CON-04-P, 2008-04EXP-01-C, 2009-01COM-03-2C, 2010-02CON-21-C, 2011-02CON-06-C, 2012-01EXP-01-2C, 2013-07CON-03-4C, and 2013-12EXP-03-5C) and all participants provided informed consent.

**Figure 1:**
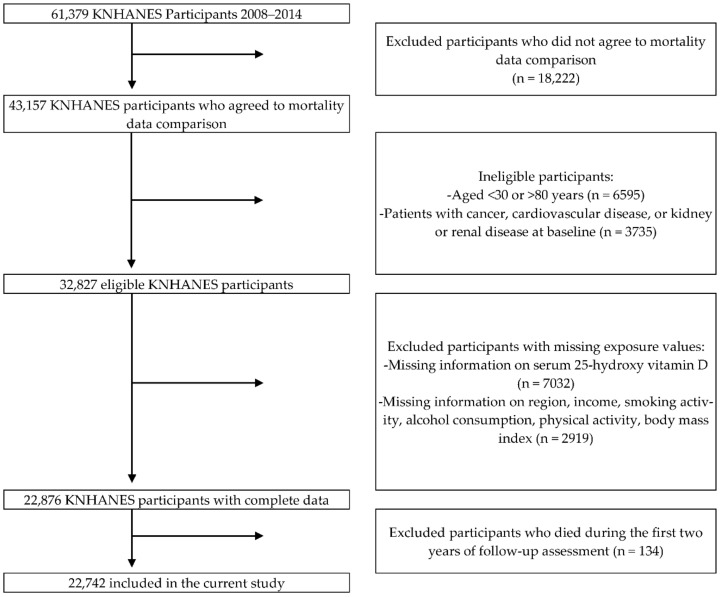
Study participant flow chart.

Among the 61,379 KNHANES participants, 43,157 (70.3%) agreed to mortality follow-up. Participants who did not agree to this were excluded from this study, but the results are not biased as a result because the excluded participants had similar demographic characteristics to those who were included. Participants aged <30 years or >80 years (n = 6595), those who were diagnosed with liver or kidney disease, cancer, or CVD at baseline (n = 3735), or whose serum 25-hydroxy vitamin D (25(OH)D) (n = 7032) and covariates (n = 2919) information was lacking, were also excluded. Finally, to reduce the possibility of reverse causation by subclinical diseases, we excluded subjects that died during the first 2 years of follow-up assessment (n = 134). Ultimately, 22,742 individuals (9325 men and 13,417 women) were included in this study (Figure 1).

### 2.2. Serum 25(OH)D Assessment

Levels of serum 25(OH)D were measured in fasting serum samples by radioimmunoassay (25-hydroxy-vitamin D125 I RIA Kit; DiaSorin, Stillwater, MN, USA). Subjects were divided into three groups: sufficient (≥20 ng/mL, following the 2011 National Academy of Medicine guidelines [29]), insufficient (12–19 ng/mL), and deficient (<12 ng/mL). For sensitivity analysis, additional cutoffs for 25(OH)D (10 and 20, 12 and 16, 12 and 30 ng/mL) were also employed.

### 2.3. Mortality Assessment

The death records of study subjects were confirmed by reviewing death certificates and medical records. A total of 1070 subjects (632 men and 438 women) had died. The causes of death were classified according to the codes of the International Classification of Diseases, 10th version (ICD-10). In this study, three causes of death were considered the endpoints of analysis: all-cause mortality, cancer mortality (C00–D48) (345 deaths), and cardiovascular disease mortality (I00–I99) (224 deaths). External deaths (S00-T98/V01-Y98) were excluded for sensitivity analyses.

### 2.4. Other Characteristics

Baseline information on participants’ demographic characteristics and lifestyle was identified through interviews with them on their health. Subjects reported on their education, household income, alcohol consumption history (never, at least once), smoking status (never, former, current), and physical activity. Household income was categorized in quartiles (<1,400,000 KRW; 1,400,000–2,670,000 KRW; 2,680,000–4,166,000 KRW; and ≥4,167,000 KRW). Metabolic equivalent task (MET) status was calculated on the basis of average frequency (days per week) and duration (hours per day) of physical activity. The MET formula assigned 2.4 METs to walking, 5.0 METs to moderate-intensity activities, and 7.5 METs to vigorous-intensity activities. Biochemical and clinical profiles regarding obesity, hypertension, and diabetes mellitus were collected during a health examination. Following standardized protocols, all health examination procedures were performed by trained medical personnel and all equipment was calibrated periodically. Hypertension was defined on the basis of systolic blood pressure of ≥140 mmHg, diastolic blood pressure of ≥90 mmHg, or hypertension medication consumption. Diabetes mellitus was defined on the basis of a fasting blood glucose level of ≥126 mg/dL, use of diabetes medication, or the reported diagnosis of diabetes. Subjects with a body mass index of ≥25.0 kg/m^2^ were classified as obese according to the Asia-Pacific regional guidelines of the WHO and the International Obesity Task Force [30].

### 2.5. Statistical Analysis

All the statistical analyses were performed using Stata MP 13 (Stata Corp LP, College Station, TX, USA) and R 3.6.1 (Vienna, Austria). Differences in participants’ characteristics were evaluated using one-way analysis of variance (ANOVA) for continuous variables and the chi-squared test for categorical variables. To explore the relationship between baseline 25(OH)D and all-cause and cause-specific mortality, we estimated the hazard ratio (HR) and the 95% confidence interval (CI) using the Cox proportional hazard model. We used age (in months) as the time metric, stratifying by the calendar year of the various KNHANES surveys. Subjects’ health histories were analyzed from the year of their involvement in the KNHANES survey (2008–2014) to their date of death or to the end of their follow-up assessment, up to 31 December 2019, whichever occurred first. All analyses accounted for sampling weights and complex survey design (cluster, strata) [28,31]. Adjustments were made for various potential confounders including sex, age, education, household income, residential area, season of blood draw, body mass index, physical activity, smoking status, and alcohol consumption. Participants were also stratified by sex or blood pressure status in order to assess whether the association between low vitamin D status and mortality varied by sex or the presence of hypertension. A *p*-value of <0.05 was considered significant.

## 3. Results

During follow-up assessment, 22,742 KNHANES subjects’ person-years amounted to a total of 202,950 and their deaths amounted to 1070 (345 cancer deaths and 224 CVD deaths). Eighty-two percent of CVD deaths were attributed to cerebrovascular disease, ischemic heart disease, and other heart diseases. Participants with a lower level of serum 25(OH)D were more likely to be female, younger, have higher education status, and live in urban areas (Table 1; sex-stratified characteristics are provided in Supplementary Table S1). Those with sufficient serum 25(OH)D comprised a higher percentage of participants that drank alcohol, were obese, or had hypertension or diabetes.

Compared with participants with sufficient vitamin D (≥20 ng/mL) and those with lower 25(OH)D were at dose-dependent higher risk of all-cause, cancer, and cardiovascular mortality (HR for adults with 25(OH)D < 12 ng/mL: 1.71 (95% CI = 1.32–2.22), 1.83 (95% CI = 1.17–2.87), and 1.72 (95% CI = 1.01–2.93), respectively) (Table 2). When stratified by sex, the negative association between vitamin D status and mortality was evident in men, but no such association was found in women (Table 2). When stratified by the presence of hypertension, the HR for all-cause mortality significantly increased in hypertensive adults with 25(OH)D below 20 ng/mL (HR: 1.46 [95% CI: 1.15–1.86] and 1.74 [95% CI: 1.24–2.44] for adults 12 ≤ 25 (OH)D < 20 ng/mL and 25(OH)D < 12 ng/mL, respectively; Table 3) and in non-hypertensive adults with 25(OH)D below 12 ng/mL (HR: 1.71 [95% CI: 1.13–2.58]). The risk of cancer and cardiovascular mortality significantly increased alongside decreasing vitamin D status only in those with hypertension (Table 3). The results were similar when using other cutoff values of 25(OH)D to categorize vitamin D status (Supplementary Table S2) and after excluding deaths due to external causes (data not shown).

## 4. Discussion

Using data from a national cohort study, we found that a low level of serum 25(OH)D is associated with an increased risk of cancer, cardiovascular, and all-cause mortality in Koreans. Men with serum 25(OH)D of <20 ng/mL had a higher risk of cancer, cardiovascular, and all-cause mortality than men with higher vitamin D status. However, no such association was observed in women. Negative relationships between vitamin D status and cancer, cardiovascular, and all-cause mortality were observed in adults with hypertension. Vitamin D status was not associated with the risk of cancer or cardiovascular mortality in non-hypertensive adults.

With the lack of RCTs conducted on Asians, the differing results between our study and recent RCTs in Western countries may be due to differences in study participants’ baseline vitamin D status, length of follow-up assessment, race, or other confounding environmental factors. The increase in the risk of all-cause, cancer, and cardiovascular mortality on the basis of lower vitamin D status shown by the current study corresponds to the results of previous observational studies [32,33,34]. However, the results of various RCTs do not support the hypothesis that vitamin D supplementation contributes to the prevention of cancer or cardiovascular mortality [14,15,16]. One explanation for this is the differences in the range of vitamin D status seen in various RCTs. In observational studies, greater mortality risk was seen in those with 25(OH)D lower than 20 ng/mL [32,33,34]. On the other hand, participant baseline vitamin D status was greater than 20 ng/mL in recent RCTs [14,15,16]. Through sensitivity analyses, we repeatedly observed a higher risk of mortality when 25(OH)D was below 16 or 20 ng/mL, but no difference in cancer mortality in adults with 25(OH)D ≥ 30 ng/mL nor in those in the range of 12–29 ng/mL (Supplementary Table S2). In this respect, our results do not contradict those of various RCTs, but rather imply a possible threshold vitamin D status that can prevent mortality. The combined results of observational studies and intervention trials suggest that the risk of mortality may plateau at serum 25(OH)D levels near 20 ng/mL. The differing results of our study and recent RCTs may also have been brought about by variations in follow-up. The mean follow-up period of RCTs is approximately 3–5 years [14,15,16], which is shorter than the mean follow-up period of nearly 9 years of the present study. Most RCTs have included deaths that occurred shortly after the initiation of the medical intervention, which may have nullified the observed effect of vitamin D supplementation on chronic diseases such as cancer and CVD. On the other hand, when a Vitamin D and Omega-3 Trial (VITAL) excluded the first 2 years of follow-up assessment, vitamin D supplementation was preventative of cancer mortality [14], which is similar to our results, as we also excluded deaths during the first 2 years of follow-up to avoid confounding factors. In addition, possible racial differences, such as sensitivity to vitamin D and susceptibility to cancer or CVD [35,36,37], combined with environmental factors, may have produced different results in RCTs performed on populations that have predominantly consisted of non-Hispanic Whites. For instance, Koreans generally consume more fruits and vegetables, which may prevent cancer and cardiovascular disease [38], than adults in the US [39]. In addition, in Korea the rates of excessive weight and obesity, which are risk factors for cancer, CVD, and mortality, are rising, but they are not as high as in the US or other European countries [40]. Still, despite its observational nature, our study cannot determine the causal effect involved, and instead it highlights the need for RCTs focused on Koreans.

The results of the previous two studies performed on Asians may seem to contradict those of this study. However, the Mendelian randomization study on Chinese participants, which found no relationship between 25(OH)D and risk of CVD or mortality, assessed participants with above 20 ng/mL [26]. Thus, as mentioned above, those results are compatible with our observational results, which show that there may be a plateau level of 25(OH)D in relation to mortality. One study in rural China, which entailed 24 years of follow-up assessment, found no association between vitamin D status and all-cause, cancer, and CVD mortality [27]. In that study, blood sampling was performed in spring when vitamin D status is lowest, and thus its results may not represent the participants’ seasonal variation in vitamin D status throughout the year, as the majority of the participants were farmers. In the present study, most participants resided in urban areas, and there was year-round recruitment of them. After adjustment for residential area and season, we found that vitamin D status was negatively associated with risk of mortality. In addition, as we excluded cancer and CVD at baseline, our results are based on a combination of the incidence of cancer and CVD, the capacity to recuperate, and mortality, across a relatively short period. The Chinese study involved follow-up over a longer period of time and may therefore have been able to observe the relapse of cancer or cardiovascular events and related deaths. Therefore, the differing participant characteristics and study designs may explain the varied results of the present study and the previous studies on Chinese participants.

Blood pressure may be an effect modifier on the risk of cancer and cardiovascular mortality by vitamin D status. Similar to the results of the present study, higher vitamin D status (≥29 ng/mL) has been associated with a lower risk of CVD and mortality in US adults with hypertension, despite the shorter follow-up period [41]. In addition, the risk of cancer mortality decreased with vitamin D supplementation in the VITAL study, in which approximately half of the participants were being treated for hypertension, when the first 2 years of follow-up were excluded [14]. As more than 40% of our study participants were hypertensive, our results are in line with previous studies in this respect, and this may explain the variations among other studies that did not account for blood pressure. In the previously mentioned Mendelian randomization study on Chinese participants, vitamin D status was not associated with cancer or cardiovascular mortality, possibly due to the low number (~16%) of hypertensive participants [26]. On the other hand, in hypertensive Chinese participants, 25(OH)D below 15.1 ng/mL was associated with greater risk of cancer incidence than seen in those with higher levels [42]. It is known that hypertension is a risk factor for cancer and cancer mortality, in addition to CVD [18,43]. However, the interaction between hypertension, vitamin D, and cancer is not clear. Vitamin D status is negatively associated with inflammatory markers in observational studies [44], but the results of RCTs are mixed in this respect [45,46,47,48]. Although blood pressure and type 2 diabetes mellitus in healthy adults were not affected by vitamin D supplementation in some RCTs [49], those with hypertension and low vitamin D status may benefit from vitamin D supplementation [50]. As adults with hypertension are already at higher risk of CVD and, thus, cardiovascular mortality, higher vitamin D status may partially prevent the worsening of cardiovascular health and the risk of cancer by lowering serum cholesterol, triglycerides, and blood pressure [26,41,49,51]. On the other hand, the benefit of vitamin D on serum lipid profiles may be minimal in adults without hypertension, resulting in a null effect on cardiovascular or cancer mortality among them. Further RCTs, such as The International Polycap Study-3, which recruited Asians with intermediate risk of CVD, may increase our understanding of the effect of vitamin D on CVD events, cancers, and cardiovascular mortality in Asians [52].

The relationship between vitamin D and mortality is evident in men, but not women, although this difference has not been fully explained. Most studies have been performed on both sexes and were adjusted for, but not stratified by sex, which may explain the incompatible results of previous studies [15] and the present study. In addition, an individual’s sex seems to be a stronger modifier than blood pressure on the relationship between vitamin D and mortality, as in the current study no association between the two was observed even in women with hypertension (data not shown), despite the higher rate of hypertensive women compared to men. Estrogen is known to decrease the risk of CVD but most women in our study were postmenopausal. Therefore, the difference between the two sexes in this respect may derive from non-hormonal causes. Although adjustment was made for smoking status, men in this study had a much higher rate of smoking than women, which may lead to higher risk of mortality, and possibly greater sensitivity to vitamin D. Smoking itself does not seem to alter serum 25(OH)D status but may affect serum activation and other biological reactions downstream of 25(OH)D [53], especially if 25(OH)D is low. Men with low vitamin D status may benefit from vitamin D supplementation in terms of decreasing their risk of mortality.

This study has some limitations. First, for each participant we used a single blood sample to assess baseline vitamin D status, which may not have reflected their long-term vitamin D status or changes in this status. Possible confounding factors were minimized by adjusting for the month of blood draw. Second, this study had a relatively short follow-up assessment period (mean 8.9 years). Consequently, we cannot completely exclude the possibility of reverse causation due to subclinical disease status. However, we excluded participants with CVD, cancer, and liver or renal disease at baseline, and those that died during the first 2 years of KNHANES follow-up assessment. More studies with longer follow-up assessment periods are needed for this population. Third, given the limited sample size and number of deaths, we were unable to distinguish the specific types of cardiovascular event or cancer that led to death. Future analyses of the ongoing KNHANES may overcome this limitation. Still, to the best of our knowledge, the present study is one of the few to retrospectively examine the association between serum 25(OH)D and mortality in an Asian population, and the first to use a nationally representative sample of Asians. Our findings were consistent in relation to multiple sensitivity analyses using various cutoffs and excluding extrinsic deaths.

## 5. Conclusions

In summary, low levels of serum 25(OH)D (<20 ng/mL) were associated with increased risk of cancer, cardiovascular, and all-cause mortality in Korean adults, especially men and adults with hypertension. Studies of Koreans, a population with low vitamin D status, are required to assess the possible causality of vitamin D status on mortality.

## Figures and Tables

**Table 1:** Baseline characteristics of study population by level of serum 25(OH)D.

Levels of Serum 25(OH)D (ng/mL)	0–12 (N = 3713)	12–20 (N = 11,192)	≥20 (N = 7837)	*p*-Value
Mean follow-up (years)	8.7 ± 0.1 ^a^	8.7 ± 0.03 ^a^	9.0 ± 0.04 ^b^	<0.001
Survey season (%) Spring (March–May) Summer (June–Aug) Autumn (Sept–Nov) Winter (Dec–Feb)	36.6 14.0 13.5 35.9	27.9 23.9 23.1 25.1	14.9 36.4 34.5 14.2	<0.001
Mean serum 25(OH)D (ng/mL)	9.9 ± 0.04 ^a^	15.9 ± 0.03 ^b^	25.4 ± 0.1 ^c^	<0.001
Age (years)				<0.001
30–44	52.2	48.0	35.9	<0.001
45–59	31.5	36.2	40.6	
60–79	16.3	15.7	23.6	
Males (%, N)	26.4 (979) ^a^	38.1 (4265) ^b^	52.1 (4081) ^c^	<0.001
Education (%) Less than high school High school graduate University	26.0 39.1 34.9	27.9 36.8 35.3	37.8 33.9 28.3	<0.001
Household income (%) Lowest Lower middle Upper middle Highest	14.2 27.1 30.4 28.3	12.8 26.0 31.1 30.1	16.0 25.7 28.8 29.5	<0.001
Urban dweller (%, N)	87.5 (3159) ^c^	83.0 (8934) ^b^	72.0 (5243) ^a^	<0.001
Lifestyle				
Smoking status (%) Never Former Current	61.7 10.0 28.3	54.7 14.4 30.9	45.6 16.6 37.8	<0.001
Current drinker (%, N)	71.0 (2396) ^a^	76.9 (7900) ^b^	79.0 (5633) ^c^	<0.001
Mean METs	1736.8 ± 67.9 ^a^	2215.1 ± 45.9 ^b^	2988.9 ± 78.8 ^c^	<0.001
Health status				
Obese (%, N)	30.1 (1089) ^a^	34.9 (3752) ^b^	35.4 (2655) ^c^	<0.001
Hypertension (%, N)	26.9 (1063) ^a^	27.0 (3266) ^a^	32.4 (2761) ^b^	<0.001
Diabetes mellitus (%, N)	8.4 (336) ^a^	8.7 (1029) ^a^	9.7 (834) ^b^	<0.001
Biochemistry				
SBP (mmHg)	117.6 ± 0.4	117.9 ± 0.2	120.1 ± 0.3	<0.001
DBP (mmHg)	76.8 ± 0.3	77.5 ± 0.1	78.3 ± 0.2	<0.001
Glucose (mg/dL)	97.6 ± 0.5	98.5 ± 0.3	98.8 ± 0.3	<0.001
TG (mg/dL)	145.8 ± 2.8	143.3 ± 1.6	141.5 ± 1.6	0.020
TC (mg/dL)	189.2 ± 0.8	191.8 ± 0.4	192.6 ± 0.5	0.212
HDL-C (mg/dL)	48.7 ± 0.3	49.0 ± 0.1	48.5 ± 0.2	<0.001
LDL-C (mg/dL)	117.9 ± 1.5	116.5 ± 0.8	117.2 ± 1.1	0.003

Values are expressed as the mean ± standard error for continuous variables and the percentage (number of counts) for categorical variables. Statistical differences among serum 25(OH)D categories were determined using the general linear model for continuous variables and the chi-square test for categorical variables. Post-hoc analyses were conducted by Bonferroni test. Superscripted letters indicate that values within a row without a common letter differ (*p* < 0.05). Household income was categorized into quartiles (lowest: <1,400,000 KRW; lower-middle: 1,400,000–2,670,000 KRW; upper middle: 2,680,000–4,166,000 KRW; and highest: ≥4,167,000 KRW). 25(OH)D, 25-hydroxyvitamin D; DBP, diastolic blood pressure; HDL-C, high-density lipoprotein cholesterol; LDL-C, low-density lipoprotein cholesterol; MET, metabolic task equivalent; SBP, systolic blood pressure; TC, total cholesterol; TG, triglycerides.

**Table 2:** Multivariable-adjusted hazard ratios (HRs) and 95% confidence intervals (CIs) for the association of serum 25(OH)D with all-cause mortality and cause-specific mortality, stratified by sex.

Serum 25(OH)D (ng/mL)	Total				Male				Female			
	Deaths/PY	Weighted Deaths/PY	HR (95% CI)	*p*-Value	Deaths/PY	Weighted Deaths/PY	HR (95% CI)	*p*-Value	Deaths/PY	Weighted Deaths/PY	HR (95% CI)	*p*-Value
All-cause mortality												
≥20	455/71,323	218,715/51,438,932	1.00 (ref)		303/36,761	151,351/31,744,736	1.00 (ref)		152/34,562	67,364/19,694,196	1.00 (ref)	
12–19	442/98,826	255,061/75,185,216	1.28 (1.06–1.54)	0.009	247/36,913	157,288/36,093,644	1.44 (1.13–1.83)	0.003	195/61,913	97,773/39,091,571	1.02 (0.77–1.34)	0.896
<12	173/32,801	101,864/25,120,105	1.71 (1.32–2.22)	<0.001	82/8253	53,555/8,823,540	2.08 (1.44–2.99)	<0.001	91/24,549	48,310/16,296,565	1.26 (0.89–1.77)	0.196
Cancer mortality												
≥20	159/71,323	77,283/51,438,932	1.00 (ref)		106/36,761	51,736/31,744,736	1.00 (ref)		53/34,562	25,548/19,694,196	1.00 (ref)	
12–19	161/98,826	92,325/75,185,216	1.36 (0.99–1.87)	0.058	91/36,913	60,380/36,093,644	1.64 (1.10–2.45)	0.016	70/61,913	31,945/39,091,571	0.93 (0.58–1.50)	0.773
<12	55/32,801	34,962/25,120,105	1.83 (1.17–2.87)	0.008	26/8253	16,814/8,823,540	1.90 (1.03–3.52)	0.041	29/24,549	18,149/16,296,565	1.47 (0.79–2.73)	0.222
Cardiovascular mortality												
≥20	90/71,323	39,489/51,438,932	1.00 (ref)		49/36,761	22,885/31,744,736	1.00 (ref)		41/34,562	16,604/19,694,196	1.00 (ref)	
12–19	93/98,826	49,866/75,185,216	1.29 (0.86–1.94)	0.218	43/36,913	25,122/36,093,644	1.80 (1.01–3.23)	0.046	50/61,913	24,744/39,091,571	0.87 (0.50–1.50)	0.618
<12	41/32,801	20,824/25,120,105	1.72 (1.01–2.93)	0.046	18/8253	10,033/8,823,540	2.96 (1.28–6.84)	0.011	23/24,549	10,791/16,296,565	1.06 (0.55–2.04)	0.852

The HRs and 95% CIs were calculated using a Cox proportional hazard regression model after adjusting for age, sex, region, income, smoking status, alcohol consumption, physical activity, and body mass index. 25(OH)D, 25-hydroxyvitamin D; PY, Person-year.

**Table 3:** Multivariable-adjusted hazard ratios (HRs) and 95% confidence intervals (CIs) for the association of serum 25(OH)D with all-cause, cancer, and cardiovascular mortality stratified by hypertension status.

Serum 25(OH)D (ng/mL)	No Hypertension				Hypertension			
	Deaths/PY	Weighted Deaths/PY	HR (95% CI)	*p*-Value	Deaths/PY	Weighted Deaths/PY	HR (95% CI)	*p*-Value
All-cause mortality								
≥20	211/45,317	108,690/34,204,328	1.00 (ref)		231/25,010	102,253/16,339,236	1.00 (ref)	
12–19	191/68,123	109,793/53,624,356	1.15 (0.87–1.52)	0.328	235/28,685	134,893/19,651,392	1.46 (1.15–1.86)	0.002
<12	66/22,867	43,152/18,024,097	1.71 (1.13–2.58)	0.011	97/9276	55,236/6,489,899	1.74 (1.24–2.44)	0.001
Cancer mortality								
≥20	82/45,317	42,013/34,204,328	1.00 (ref)		75/25,010	33,557/16,339,236	1.00 (ref)	
12–19	68/68,123	42,655/53,624,356	1.10 (0.70–1.74)	0.668	89/28,685	47,100/19,651,392	1.74 (1.12–2.72)	0.015
<12	23/22,867	15,357/18,024,097	1.52 (0.78–2.98)	0.222	32/9276	19,605/6,489,899	2.27 (1.22–4.22)	0.010
Cardiovascular mortality								
≥20	39/45,317	17,135/34,204,328	1.00 (ref)		48/25,010	20,785/16,339,236	1.00 (ref)	
12–19	28/68,123	12,317/53,624,356	0.82 (0.39–1.73)	0.605	60/28,685	34,857/19,651,392	1.75 (1.08–2.85)	0.023
<12	<10/22,867	3742/18,024,097	1.01 (0.33–3.11)	0.982	29/9276	15,372/6,489,899	2.23 (1.17–4.24)	0.015

The HRs and 95% CIs were calculated using a Cox proportional hazard regression model after adjusting for age, sex, region, income, smoking status, alcohol consumption, physical activity, and body mass index. 25(OH)D, 25-hydroxyvitamin D; PY, Person-year.

## Data Availability

Restrictions apply to the availability of these data. Data were obtained from the Korea Centers for Disease Control and Prevention and are available with the permission of this agency.

## Supplementary Materials

The following supporting information can be downloaded at: https://www.mdpi.com/article/10.3390/nu14091849/s1, Table S1: Baseline characteristics of study population by level of serum 25(OH)D and sex; Table S2: Multivariable-adjusted hazard ratios (HRs) and 95% confidence intervals (CIs) for the association of serum 25(OH)D with all-cause mortality and cause-specific mortality using various 25(OH)D thresholds, stratified by sex.
