# Supplementary material for: Low Vitamin D Status Is Associated with Increased Risk of Mortality in Korean Men and Adults with Hypertension: A Population-Based Cohort Study
Source: Nutrients. 2022 Apr 28;14(9):1849. doi: 10.3390/nu14091849 (PMC9105830; doi:10.3390/nu14091849)
Supplement: Supplementary file 1 [file nutrients-14-01849-s001.zip › nutrients-1631895-supplementary.pdf]

nutrients MDPI

Supplementary Table S1. Baseline characteristics of study population by level of serum 25(OH)D and sex

Sex	Males				Females			
Levels of serum 25(OH)D (ng/mL)	0 – 12 (N = 979)	12 – 20 (N = 4,265)	≥ 20 (N = 4,081)	p-Value	0 – 12 (N = 2,734)	12 – 20 (N = 6,927)	≥ 20 (N = 3,756)	p-Value
Mean follow-up (years)	8.6±0.1[a]	8.7±0.04[a]	9.1±0.1[b]	<0.001	8.9±0.1[a]	8.9±0.03[a]	9.1±0.1[b]	<0.001
Survey season (%)								
Spring (March – May)	35.9	30.2	16.1	<0.001	37.1	25.7	12.9	<0.001
Summer (June – Aug)	14.1	21.9	36.1		13.9	25.8	36.9	
Autumn (Sept – Nov)	11.8	20.7	33.7		14.5	25.4	35.9	
Winter (Dec-Feb)	38.2	27.2	14.1		34.5	23.1	4.3	
Mean serum 25(OH)D (ng/mL)	10.0±0.1[a]	16.1±0.04[b]	25.6±0.1[c]	<0.001	9.8±0.04[a]	15.7±0.04[b]	25.2±0.1[c]	<0.001
Age (years)								
30-44	55.4	51.4	38.3	<0.001	50.4	44.8	32.0	<0.001
45-59	31.8	35.3	41.7		31.4	37.2	38.6	
60-79	12.8	13.3	19.9		18.2	18.1	29.4	
Education (%)								
Less than high school	17.9	20.2	31.1	<0.001	31.5	35.1	48.4	<0.001
High school graduate	37.2	36.1	35.4		40.3	37.4	31.5	
University	44.9	43.6	33.5		29.1	27.5	20.1	
Household income (%)								
Lowest	12.3	10.5	13.0	0.031	15.4	15.1	20.7	<0.001
Lower middle	27.0	25.2	25.5		27.1	26.7	26.0	
Upper middle	32.7	32.6	30.0		29.1	29.7	27.0	
Highest	28.0	31.7	31.5		28.4	28.5	26.2	
Urban dweller (%, N)	90.2 (861)[c]	83.7 (3,455)[b]	70.7 (2,680)[a]	<0.001	86.0 (2,298)[c]	82.4 (5,479)[b]	74.0 (2,563)[a]	<0.001
Lifestyle								
Smoking status (%)								
Never	13.3	17.7	17.0	0.004	89.2	89.3	91.4	0.012
Former	22.0	25.5	25.3		3.3	4.1	2.7	
Current	64.8	56.9	57.7		7.6	6.6	5.8	
Current drinker (%, N)	85.1 (787)[a]	87.3 (3,538)[a]	87.2 (3,369)[a]	0.245	63.0 (1,609)[a]	67.2 (4,362)[b]	65.7 (2,264)[ab]	0.006
Mean METs	1974.8±116.6[a]	2449.3±73.5[b]	3319.7±98.5[c]	<0.001	1600.8±81.6[a]	1995.9±49.5[b]	2454.2±93.1[c]	<0.001
Health status								
Obesity (%, N)	35.2 (326)[a]	40.6 (1,647)[b]	38.1 (1,458)[a]	0.002	27.2 (763)[a]	29.5 (2,105)[b]	31.0 (1,197)[b]	0.071
Hypertension (%, N)	31.7 (335)[ab]	31.4 (1,486)[a]	34.9 (1,548)[b]	0.101	24.0 (728)[a]	22.9 (1,780)[a]	28.4 (1,213)[b]	<0.001
Diabetes (%, N)	10.2 (112)[a]	9.9 (486)[a]	10.3 (477)[a]	0.306	7.4 (224)[a]	7.6 (543)[a]	8.8 (357)[b]	0.006
Biomarker								
SBP (mmHg)	121.1±0.7[ab]	120.2±0.3[a]	121.6±0.3[b]	<0.001	115.6±0.4[a]	115.8±0.3[a]	117.7±0.4[b]	<0.001
DBP (mmHg)	80.7±0.5[a]	80.5±0.2[a]	80.4±0.2[a]	<0.001	74.6±0.3[a]	74.6±0.2[a]	74.8±0.2[a]	<0.001
Glucose (mg/dL)	100.2±1.1[a]	100.6±0.4[a]	100.0±0.4[a]	<0.001	96.1±0.5[a]	96.5±0.3[a]	96.8±0.4[a]	<0.001
TG (mg/dL)	191.4±6.7[c]	172.1±2.8[b]	158.3±2.4[a]	<0.001	119.7±2.0[b]	115.8±1.4[ab]	114.1±1.4[a]	<0.001
TC (mg/dL)	190.9±1.6[a]	192.8±0.6[a]	191.9±0.7[a]	<0.001	188.3±0.8[a]	190.9±0.5[b]	193.9±0.7[c]	<0.001
HDL-C (mg/dL)	45.1±0.5[a]	46.1±0.2[b]	46.6±0.2[b]	<0.001	50.8±0.3[a]	51.8±0.2[b]	51.5±0.2[b]	<0.001
LDL-C (mg/dL)	120.0±2.3[a]	118.1±1.2[a]	116.0±1.4[a]	0.001	115.6±1.9[ab]	114.4±1.1[a]	119.6±1.6[b]	0.614

Values are expressed as the mean ± standard error for continuous variables and the percentage (number of counts) for categorical variables. Statistical differences among serum 25(OH)D categories were determined using the general linear model for continuous variables and the chi-square test for categorical variables. Post-hoc analyses were conducted by Bonferroni test. Superscripted

letters indicate that values within a row without a common letter differ ($p < 0.05$). Household income was categorized into quartiles (lowest: < 1,400,000 KRW; lower-middle: 1,400,000 – 2,670,000 KRW; upper middle: 2,680,000-4,166,000 KRW; and highest: ≥4,167,000 KRW).25(OH)D, 25-hydroxyvitamin D; DBP, diastolic blood pressure; HDL-C, high-density lipoprotein cholesterol; LDL-C, low-density lipoprotein cholesterol; MET, metabolic task equivalent; SBP, systolic blood pressure; TC, total cholesterol; TG, triglycerides.

Supplementary Table S2. Multivariable-adjusted hazard ratios (HRs) and 95% confidence intervals (CIs) for the association of serum 25(OH)D with all-cause mortality and cause-specific mortality using various 25(OH)D thresholds, stratified by sex

Serum 25(OH)D (ng/mL)	Total				Male				Female			
	Death/PY	Weighted Death/PY	HR (95% CI)	p-value	Death/PY	Weighted Death/PY	HR (95% CI)	p-value	Death/PY	Weighted Death/PY	HR (95% CI)	p-value
All-cause mortality												
≥20	455/71,323	218,715/51,438,932	1.00 (ref)		303/36,761	151,351/31,744,736	1.00 (ref)		152/34,562	67,364/19,694,196	1.00 (ref)	
10-19	529/117,271	310,295/89,128,720	1.32 (1.10-1.58)	0.003	288/41,872	185,827/41,341,805	1.48 (1.17-1.87)	0.001	241/75,399	124,468/47,786,915	1.05 (0.81-1.37)	0.706
<10	86/14,356	46,631/11,176,601	1.88 (1.35-2.61)	<0.001	41/3,294	25,015/3,575,379	2.34 (1.49-3.68)	<0.001	45/11,062	21,616/7,601,221	1.31 (0.82-2.08)	0.252
Cancer mortality												
≥20	159/71,323	77,283/51,438,932	1.00 (ref)		106/36,761	51,736/31,744,736	1.00 (ref)		53/34,562	25,548/19,694,196	1.00 (ref)	
10-19	186/117,271	110,930/89,128,720	1.41 (1.03-1.92)	0.033	103/41,872	68,740/41,341,805	1.66 (1.11-2.46)	0.013	83/75,399	42,190/47,786,915	1.01 (0.63-1.61)	0.974
<10	30/14,356	16,358/11,176,601	1.96 (1.09-3.53)	0.025	14/3,294	8,455/3,575,379	2.03 (0.92-4.49)	0.081	16/11,062	7,903/7,601,221	1.57 (0.69-3.57)	0.286
Cardiovascular mortality												
≥20	90/71,323	39,489/51,438,932	1.00 (ref)		49/36,761	22,885/31,744,736	1.00 (ref)		41/34,562	16,604/19,694,196	1.00 (ref)	
10-19	121/117,271	65,416/89,128,720	1.40 (0.95-2.05)	0.088	56/41,872	33,630/41,341,805	2.05 (1.18-3.56)	0.011	65/75,399	31,786/47,786,915	0.91 (0.55-1.52)	0.729
<10	13/14,356	5,274/11,176,601	1.05 (0.48-2.27)	0.905	<10/3,294	1,525/3,575,379	0.73 (0.23-2.30)	0.589	<10/11,062	3,749/7,601,221	0.96 (0.37-2.48)	0.938
All-cause mortality												

≥16	667/119,935	342,791/88,182,049	1.00 (ref)		424/57,213	228,556/50,920,199	1.00 (ref)		243/62,722	114,235/37,261,850	1.00 (ref)	
12-15	230/50,214	130,985/38,442,099	1.29 (1.04-1.60)	0.022	126/16,461	80,083/16,918,182	1.60 (1.19-2.16)	0.002	104/33,752	50,902/21,523,917	0.93 (0.69-1.24)	0.608
<12	173/32,801	101,864/25,120,105	1.60 (1.24-2.05)	<0.001	82/8,253	53,555/8,823,540	1.94 (1.37-2.76)	<0.001	91/24,549	48,310/16,296,565	1.21 (0.87-1.67)	0.259
Cancer mortality												
≥16	238/119,935	123,509/88,182,049	1.00 (ref)		152/57,213	83,662/50,920,199	1.00 (ref)		86/62,722	39,847/37,261,850	1.00 (ref)	
12-15	82/50,214	46,100/38,442,099	1.38 (0.98-1.93)	0.064	45/16,461	28,454/16,918,182	1.71 (1.07-2.73)	0.026	37/33,752	17,646/21,523,917	0.98 (0.60-1.59)	0.921
<12	55/32,801	34,962/25,120,105	1.68 (1.13-2.52)	0.011	26/8,253	16,814/8,823,540	1.67 (0.95-2.95)	0.078	29/24,549	18,149/16,296,565	1.52 (0.87-2.66)	0.139
Cardiovascular mortality												
≥16	137/119,935	63,812/88,182,049	1.00 (ref)		71/57,213	35,748/50920199	1.00 (ref)		66/62,722	28,063/37,261,850	1.00 (ref)	
12-15	46/50,214	25,544/38,442,099	1.22 (0.77-1.94)	0.391	21/16,461	12,259/16918182	1.76 (0.89-3.49)	0.105	25/33,752	13,285/21,523,917	0.88 (0.50-1.56)	0.662
<12	41/32,801	20,824/25,120,105	1.57 (0.96-2.56)	0.073	18/8,253	10,033/8823540	2.46 (1.13-5.39)	0.024	23/24,549	10,791/16,296,565	1.11 (0.61-2.02)	0.729
All-cause mortality												
≥30	113/12,516	53,430/8,423,117	1.00 (ref)		72/7,216	34,409/5,574,582	1.00 (ref)		41/5,300	19,021/2,848,535	1.00 (ref)	
12-29	784/157,633	420,346/118,200,000	1.02 (0.80-1.29)	0.897	478/66,459	274,230/62,263,798	1.27 (0.96-1.67)	0.093	306/91,175	146,116/55,937,233	0.61 (0.39-0.96)	0.031
<12	173/32,801	101,864/25,120,105	1.48 (1.06-2.05)	0.020	82/8,253	53,555/8,823,540	2.07 (1.36-3.14)	0.001	91/24,549	48,310/16,296,565	0.78 (0.47-1.32)	0.358
Cancer mortality												
≥30	39/12,516	19,006/8,423,117	1.00 (ref)		28/7,216	12,466/5,574,582	1.00 (ref)		11/5,300	6,540/2,848,535	1.00 (ref)	
12-29	281/157,633	150,602/118,200,000	0.97 (0.62-1.50)	0.880	169/66,459	99,650/62,263,798	1.13 (0.72-1.79)	0.600	112/91,175	50,952/55,937,233	0.67 (0.27-1.65)	0.381

<12	55/32,801	34,962/25,120,105	1.46 (0.83-2.56)	0.191	26/8,253	16,814/8,823,540	1.57 (0.80-3.07)	0.187	29/24,549	18,149/16,296,565	1.05 (0.39-2.86)	0.922
Cardiovascular mortality												
≥30	20/12,516	8,286/8,423,117	1.00 (ref)		10/7,216	4,111/5,574,582	1.00 (ref)		10/5,300	4,175/2,848,535	1.00 (ref)	
12-29	163/157,633	81,069/118,200,000	1.55 (0.79-3.02)	0.199	82/66,459	43,896/62,263,798	3.27 (1.15-9.32)	0.027	81/91,175	37,173/55,937,233	0.63 (0.29-1.41)	0.261
<12	41/32,801	20,824/25,120,105	2.21 (0.97-5.03)	0.058	18/8,253	10,033/8,823,540	6.35 (1.75-23.07)	0.005	23/24,549	10,791/16,296,565	0.76 (0.29-1.96)	0.570

The HRs and 95% CIs were calculated using a Cox proportional hazard regression model after adjusting for age, sex, region, income, smoking status, alcohol consumption, physical activity, and body mass index. 25(OH)D, 25-hydroxyvitamin D; PY, Person-year
